# Functional connectivity changes in adults with developmental stuttering: a preliminary study using quantitative electro-encephalography

**DOI:** 10.3389/fnhum.2014.00783

**Published:** 2014-10-13

**Authors:** Kathleen Joos, Dirk De Ridder, Ronny A. Boey, Sven Vanneste

**Affiliations:** ^1^Department of Neurosurgery, University Hospital AntwerpAntwerp, Belgium; ^2^Department of Translational Neuroscience, Faculty of Medicine, University of AntwerpAntwerp, Belgium; ^3^Department of Surgical Sciences, Dunedin School of Medicine, University of OtagoDunedin, New Zealand; ^4^BRAI^2^N and TRI, Sint Augustinus HospitalAntwerp, Belgium; ^5^Centre of Stuttering Therapy Antwerp, University of AntwerpAntwerp, Belgium; ^6^Faculty of Medicine and Health Sciences, University of AntwerpAntwerp, Belgium; ^7^School of Behavioral and Brain Sciences, The University of Texas at DallasRichardson, TX, USA

**Keywords:** developmental stuttering, sensorimotor timing deficit, functional connectivity, qEEG, sLORETA

## Abstract

**Introduction**: Stuttering is defined as speech characterized by verbal dysfluencies, but should not be seen as an isolated speech disorder, but as a generalized sensorimotor timing deficit due to impaired communication between speech related brain areas. Therefore we focused on resting state brain activity and functional connectivity.

**Method**: We included 11 patients with developmental stuttering and 11 age matched controls. To objectify stuttering severity and the impact on quality of life (QoL), we used the Dutch validated Test for Stuttering Severity-Readers (TSS-R) and the Overall Assessment of the Speaker’s Experience of Stuttering (OASES), respectively. Furthermore, we used standardized low resolution brain electromagnetic tomography (sLORETA) analyses to look at resting state activity and functional connectivity differences and their correlations with the TSS-R and OASES.

**Results**: No significant results could be obtained when looking at neural activity, however significant alterations in resting state functional connectivity could be demonstrated between persons who stutter (PWS) and fluently speaking controls, predominantly interhemispheric, i.e., a decreased functional connectivity for high frequency oscillations (beta and gamma) between motor speech areas (BA44 and 45) and the contralateral premotor (BA6) and motor (BA4) areas. Moreover, a positive correlation was found between functional connectivity at low frequency oscillations (theta and alpha) and stuttering severity, while a mixed increased and decreased functional connectivity at low and high frequency oscillations correlated with QoL.

**Discussion**: PWS are characterized by decreased high frequency interhemispheric functional connectivity between motor speech, premotor and motor areas in the resting state, while higher functional connectivity in the low frequency bands indicates more severe speech disturbances, suggesting that increased interhemispheric and right sided functional connectivity is maladaptive.

## Introduction

Developmental stuttering is a frequently occurring disorder of verbal fluency, a speech characterized by frequent repetition or prolongation of sounds, syllables or words, or by frequent hesitations or pauses that disrupt the rhythmic flow of speech (World Health Organization, 1997). Most often it becomes apparent around the age of 3–5 years, during the phase of speech acquisition (Bloodstein, 1995). Despite the fact that nearly 80% resolves by adulthood, a prevalence of 1% remains in the adult population with a male/female ratio of 4:1 (Andrews, 1964). Besides the typical verbal dysfluencies, stuttering is often accompanied by secondary behaviors, including eye blinking, jaw jerking, head or other involuntary movements (Prasse and Kikano, 2008). The combination of stuttering and the non-speech behaviors was proven to have a high impact on emotional, physical and mental functioning (Craig et al., 2009), in addition to impaired professional and academic opportunities (Klein and Hood, 2004; Hughes et al., 2010).

Although a wide range of environmental factors are related to the onset of stuttering, including physical, emotional, behavioral and developmental factors (Conture, 2001; Paden, 2005), the etiology of stuttering is still ambiguous. Since the early 1930’s, it has been claimed that stuttering has its origin in the central nervous system. One of the most consistent findings observed in persons who stutter (PWS) is an anomalous control of the articulatory, laryngeal and respiratory system, mainly due to a timing and coordination deficit (McClean and Runyan, 2000; Kleinow et al., 2001; McClean et al., 2004; Max and Gracco, 2005; Loucks and De Nil, 2006; Loucks et al., 2007). It has become clear that stuttering is not an isolated phenomenon, but rather an element of a more generalized motor timing deficit, as demonstrated in non-speech related movements like finger flexion (Borden, 1983), finger tapping (Smits-Bandstra et al., 2006) and finger movement sequencing (Forster and Webster, 2001). This suggests an impairment of dynamic interaction between cortical and subcortical brain areas related to motor planning, initiation and execution (Watkins et al., 2008; Lu et al., 2010).

Previous research demonstrated that developmental stuttering is associated to structural anomalies near the left rolandic operculum (Sommer et al., 2002; Kell et al., 2009), while alterations of neural activity in the right hemisphere, namely nearby the right rolandic operculum, have been identified as a compensation mechanism for these fundamental structural deficiencies using positron emission tomography (PET) and functional magnetic resonance imaging (fMRI; Fox et al., 1996; Braun et al., 1997a,b; Preibisch et al., 2003). Additionally, these abnormal activation patterns normalize with fluency shaping therapies and this in combination with the activation of a more left-sided network (Neumann et al., 2005). Contrary to these observations, spontaneous recovery correlates with white matter changes in the vicinity of the left rolandic operculum, implying that the right hemisphere is not specialized enough for a complete recovery (Kell et al., 2009). In general, the most consistent observations retrieved by functional imaging during stuttered speech are: (1) increased activation of the right pars opercularis; (2) absence of activation of bilateral auditory cortices; (3) overactivation of the vermal region of the cerebellum; and (4) increased activity of the dopaminergic midbrain, mainly the substantia nigra (Brown et al., 2005).

Recently, there is an increasing interest in both structural and functional connectivity as a reversed activation of speech related areas has been observed during stuttering, i.e., initiation of the motor program before fulfilling the articulatory coding. Making use of magneto-encephalography, a delayed activation of the left inferior frontal gyrus (IFG) with an early recruitment of the left motor cortex was observed (Salmelin et al., 2000). Interestingly, this altered sequence of activation could not only be identified during overt stuttering, but also during fluent speech (Salmelin et al., 2000), suggesting a more generalized timing deficit in stuttering persons. An impaired structural connectivity, identified by diffusion tensor imaging, between the sensorimotor cortex representing the oropharynx and the left pars opercularis as well as the premotor cortex has been assumed to play an etiologic factor (Sommer et al., 2002). A recent fMRI study demonstrated both decreased functional and structural connectivity between left IFG and premotor cortex when comparing PWS with fluently speaking controls in combination with an increased functional connectivity between right pars opercularis and bilateral speech related brain areas during both speech and non-speech related tasks (Chang et al., 2011).

Currently, most research looked at structural alterations or at functional differences during speech or speech related tasks. However, it has been demonstrated that stuttering is the expression of a more generalized motor timing deficit, as PWS also show alterations in non-speech related movements (Borden, 1983; Forster and Webster, 2001; Smits-Bandstra et al., 2006), making it conceivable that intrinsic alterations in neural activity and resting state functional connectivity in and between brain areas related to planning, initiation and execution of motor tasks are present in PWS. We therefore use resting state quantitative electro-encephalography (qEEG) and standardized low resolution brain electromagnetic tomography (sLORETA) to reveal changes in neural activity and functional connectivity. The main advantage of qEEG is that it has a higher time resolution than fMRI and PET, moreover it measures brain electric activity directly, while the other methods record changes in blood flow, i.e., fMRI, or metabolic activity, i.e., PET. In addition, we do not have to cope with the noisy environment of fMRI. This study might make a significant contribution in order to understand the pathophysiology of stuttering, as it excludes speech or task related changes in brain activity because they impede the interpretation of the observed alterations in neural activity and functionality as the alterations might be the underlying cause or the consequence of stuttering.

## Methods

### Participants

Eleven patients with developmental stuttering and a mean age of 27.82 years (*SD* = 6.38) were included in the study (Table 1) as well as eleven age-matched controls, selected from a database, with a mean age of 28.00 years (*SD* = 6.83). All patients reported that they had been stuttering since childhood and all participants were screened by a physician and a speech therapist specialized in stuttering. Inclusion criteria were: (1) mild to severe stuttering based on the Test for Stuttering Severity-Readers (TSS-R); (2) age between 18 and 50 years; (3) right-handed as assessed by the Edinburgh Handedness Inventory; and (4) native Dutch speaking persons. To obtain a correlation between brain activity/connectivity and questionnaires, only eight subjects could be included as the Overall Assessment of the Speaker’s Experience of Stuttering (OASES) questionnaire was not filled in by 3 of them. The study was approved by the ethical committee of the University Hospital Antwerp. All subjects signed an informed consent. Data were not placed in a public or institutional repository, but can me be made available upon request.

**Table 1:** **Demographic and stuttering characteristics**.

Patient	Age (years)	Gender	TSS-R	OASES
1	24	male	18	61.3
2	32	male	28	56.2
3	18	male	18	47.4
4	27	male	5	42.2
5	33	male	20	39.4
6	25	male	25	/
7	19	male	13	/
8	36	male	11	41.2
9	36	male	8	39.6
10	32	male	27	51.6
11	24	male	23	/
Mean	27.82		17.82	47.36
SD	6.38		7.76	8.27

### Questionnaires

In first instance we assessed the severity of stuttering using the Dutch validated TSS-R (Boey, 2007). For the interpretation of the total scores, results were converted to percentiles, whereas a subdivision can be made in very mild (1–9), mild (10–24), mild-moderate (25–49), moderate (50–59), moderate-severe (60–74), severe (75–89) and very severe (90–100). It has to be noticed that we used the total score for further statistical analysis and not the percentiles. To evaluate the impact of stuttering on the quality of life (QoL) the OASES (Yaruss and Quesal, 2006) is used. This questionnaire classifies the impact of stuttering in five subscales: mild (20–29.9), mild-moderate (30–44.9), moderate (45–59.9), moderate-severe (60–74.9) and severe (75–100).

### EEG data collection

EEGs were obtained in a fully lighted room with each participant sitting upright in a comfortable chair. The EEG was sampled with 19 electrodes (Fp1, Fp2, F7, F3, Fz, F4, F8, T7, C3, Cz, C4, T8, P7, P3, Pz, P4, P8, O1 and O2) in the standard 10–20 International placement referenced to linked ears and impedances were checked to remain below 5 kΩ. Data were collected with the eyes closed during 5 min, from which we removed the artifacts. Subsequently, we collected the first 100 2-s epochs of the remaining EEG (sampling rate = 1024 Hz, band passed 0.15–200 Hz). Data were resampled to 128 Hz, band-pass filtered (fast Fourier transform filter) to 2–44 Hz. These data were transposed into Eureka! Software (Congedo, 2002), plotted and carefully inspected for manual and ICA dependent artifact-rejection. All episodic artifacts including eye blinks, eye movements, teeth clenching, body movement, or ECG artifacts were removed from the stream of the EEG.

### EEG power spectral analysis

**Figure 1:**
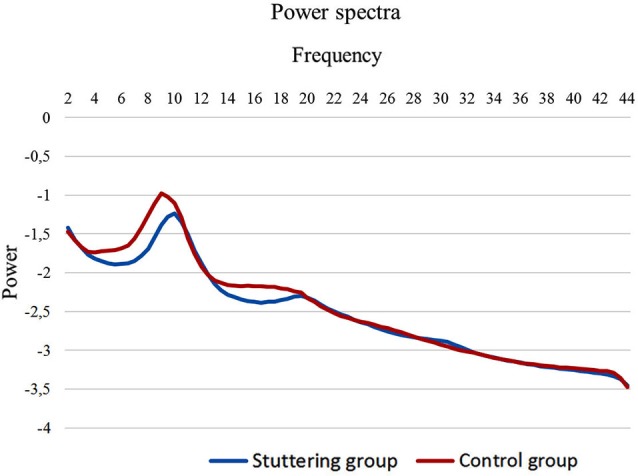
**EEG power spectra**. EEG power spectra in stuttering patients (blue line) and fluently speaking controls (red line) averaged over all electrodes.

To compute the power spectral analysis for the power density of EEG rhythms, we performed a digital FFT-based power spectrum analysis (Time Domain Tapering: Hamming, Frequency Domain Smoothing: Blackman, Overlapping FFT Windows Advancement Factor: 8) with a resolution of 0.5 Hz. In order to give an overview of the EEG data we averaged the log-transformed spectra of all 19 scalp electrodes for each subject. Subsequently, we calculated the average spectrum for the stuttering patients and the fluently speaking controls (Figure 1). Moreover, we performed a multivariate analysis of variance (MANOVA) to identify differences in power spectra for each of the eight frequency bands between PWS and controls.

### Source localization

SLORETA (Pascual-Marqui, 2002) was used to estimate the intracerebral electrical sources that generated the scalp-recorded activity in each of the eight frequency bands, i.e., delta (2–3.5 Hz), theta (4–7.5 Hz), alpha 1 (8–10 Hz), alpha 2 (10.5–12 Hz), beta 1 (12.5–18 Hz), beta 2 (18.5–21 Hz), beta 3 (21.5–30 Hz) and gamma (30.5–44 Hz). It computes electric neuronal activity as current density (A/m^2^) without assuming a predefined number of active sources. The sLORETA solution space consists of 6239 voxels (voxel size: 5–5–5 mm) and is restricted to cortical gray matter and hippocampi, as defined by digitized MNI 152 template (Fuchs et al., 2002). Scalp electrode coordinates on the MNI brain are derived from the international 10–20 system (Jurcak et al., 2007). The tomography sLORETA has received considerable validation from studies combining LORETA with other more established localization methods, such as fMRI (Vitacco et al., 2002; Mulert et al., 2004), structural MRI (Worrell et al., 2000) and PET (Dierks et al., 2000; Pizzagalli et al., 2004; Zumsteg et al., 2005). Further sLORETA validation has been based on accepting as ground truth the localization findings obtained from invasive, implanted depth electrodes, in which case there are several studies in epilepsy (Zumsteg et al., 2006a,b) and cognitive event-related potentials (Volpe et al., 2007).

### Region of interest analysis

The log-transformed electric current density was averaged across all voxels belonging to the region of interest (ROI), i.e., Broca’s area and its right sided homologue (BA44, BA45), premotor cortex (BA6) and motor cortex (BA4), separately for each frequency band. These ROI’s were defined based on previous brain research on stuttering.

### Functional connectivity

Coherence and phase synchronization between time series corresponding to different spatial locations are usually interpreted as indicators of the “functional connectivity”. However, any measure of dependence is highly contaminated with an instantaneous, non-physiological contribution due to volume conduction and low spatial resolution (Pascual-Marqui, 2007a). Therefore Pascual-Marqui introduced a new technique (i.e., Hermitian covariance matrices) that removes this confounding factor considerably (Pascual-Marqui, 2007b). As such, this measure of dependence can be applied to any number of brain areas jointly, i.e., distributed cortical networks, whose activity can be estimated with sLORETA. Measures of linear dependence (coherence) between the multivariate time series are defined. The measures are expressed as the sum of lagged dependence and instantaneous dependence. The measures are non-negative, and take the value 0 only when there is independence of the pertinent type and are defined in the frequency domains: delta (2–3.5 Hz), theta (4–7.5 Hz), alpha 1 (8–10 Hz), alpha 2 (10.5–12 Hz), beta 1 (12.5–18 Hz), beta 2 (18.5–21 Hz), beta 3 (21.5–30 Hz) and gamma (30.5–44 Hz). Based on this principle lagged linear connectivity was calculated.

### Statistical analysis

First of all we used sLORETA to identify differences in neural activity by performing a voxel-by-voxel between-groups comparison of the current density distribution. More specifically, nonparametric statistical analyses of the sLORETA images were used (statistical nonparametric mapping; SnPM) for each contrast employing a *t*-statistic for unpaired groups with a correction for multiple comparisons (*p* < 0.05). Due to the non-parametric nature of this method, this method does not have to rely on any assumption of normal distribution and it readily accounts for the multiple comparisons problem (Nichols and Holmes, 2002). One voxel-by-voxel test (comprising 6.239 voxels each) for the different frequency bands was performed.

To identify differences in the log-transformed current density between the 8 ROI’s, we performed a MANOVA for all 8 ROI’s and all frequency bands. Furthermore, we calculated the Pearson’s correlations between the log-transformed current density in the 8 ROI’s for all frequency bands and stuttering severity and OASES.

Connectivity contrast maps were calculated through multiple ROI-by-ROI comparisons using *t*-statistics. The significance threshold was based on a permutation test with 5000 permutations. Again a comparison was made between the stuttering patients vs. the fluently speaking controls. In addition the correlation was calculated between the connectivity maps and respectively stuttering severity and OASES.

## Results

### Questionnaires

All patients were evaluated with the TSS-R and eight patients filled in the OASES questionnaire. The mean score for the TSS-R was 17.82 (*SD* = 7.76), while the mean total score for the OASES was 47.36 (*SD* = 8.27), which can both be interpreted as moderate.

### Power spectra

We initially looked at the spectral analyses of both groups to demonstrate the quality of our EEG data (Figure 1). Moreover, we observed an overall significant difference between stuttering adults and controls in the spectra averaged over all electrodes (*F*_(1,20)_ = 5.65; *p* < 0.01). More specifically, power spectra differed significantly between controls and patients in the theta frequency band (*F*_(1,20)_ = 4.95; *p* < 0.01), respectively −1.65 µV^2^ (*SD* = 0.27) and −1.86 µV^2^ (*SD* = 0.16), and in the beta 1 frequency band (*F*_(1,20)_ = 5.06; *p* < 0.05), respectively −2.15 µV^2^ (*SD* = 0.21) and −2.31 µV^2^ (*SD* = 0.11).

### Neural activity: PWS vs. fluently speaking controls (whole brain analysis)

A comparison was made between all stuttering patients and fluently speaking controls. No significant results were obtained in neural activity by source localization in any of the eight frequency bands.

### Neural activity: correlation with stuttering severity and the impact on daily living (whole brain analysis)

No significant correlation could be observed between neural activity and TSS-R or OASES for any of the eight frequency bands.

### Neural activity: PWS vs. fluently speaking controls (ROI analysis)

Statistical analysis yielded significant effects between PWS and fluently speaking controls when comparing neural activity in the eight frequency bands for the eight different ROI’s, however, they could not withstand the correction for multiple corrections.

### Neural activity: correlation with stuttering severity and the impact on daily living (ROI analysis)

No significant correlation could be obtained between any of the eight frequency bands in the eight ROI’s and stuttering severity. In addition, significant correlations could be observed between neural activity and the OASES, however none of these results remained significant after correction for multiple comparisons.

### Functional connectivity: PWS vs. fluently speaking controls

**Figure 2:**
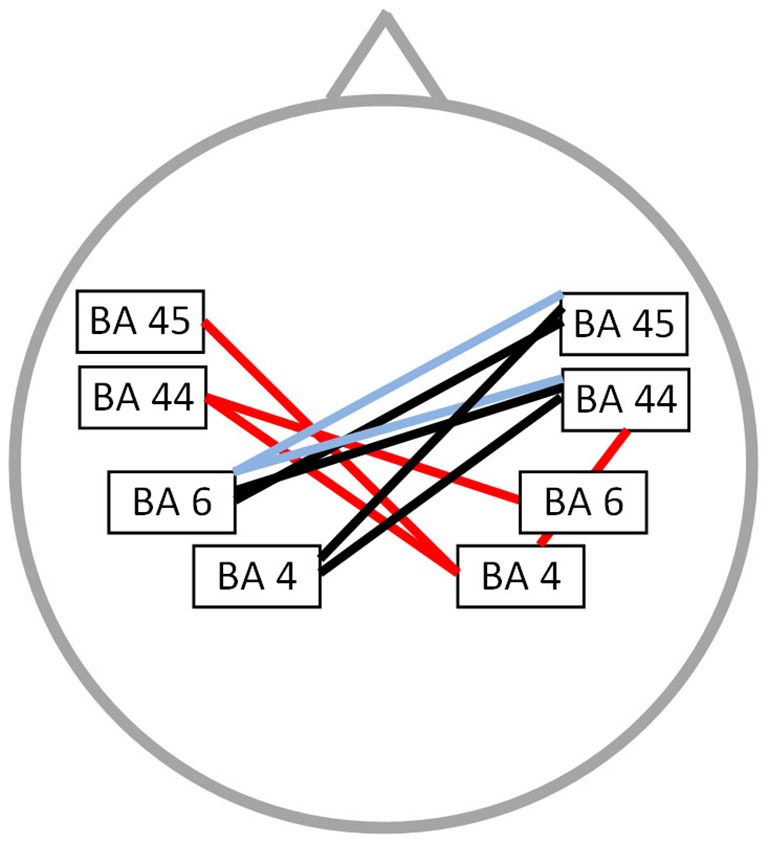
**Connectivity differences between PWS and fluently speaking controls**. Decreased functional connectivity (*p* < 0.01) in the beta 1 frequency band (red lines), beta 3 frequency band (bleu lines) and gamma frequency band (black lines) in stuttering patients vs. fluently speaking controls.

Functional connectivity analysis between PWS and fluently speaking controls yielded a significant difference for beta 1, beta 3 and gamma band activity (*t*_(1,20)_ = 2.37; *p* < 0.01) (Figure 2). A decreased connectivity could be observed for beta 1 between left pars triangularis (BA45) and opercularis (BA44), i.e., Broca’s areas, and right motor cortex, as well as between the right premotor area and left BA44 and the right pars opercularis and the right motor cortex. Furthermore, a significant decrease in connectivity for beta 3 could be observed between the left premotor and right BA44 and BA45. Decreased connectivity for the gamma frequency band is present between left motor and premotor cortex and right sided BA44 and BA45. No significant difference could be found for the delta, theta, alpha 1, alpha 2 and beta 2 frequency band.

### Functional connectivity: correlation with stuttering severity

**Figure 3:**
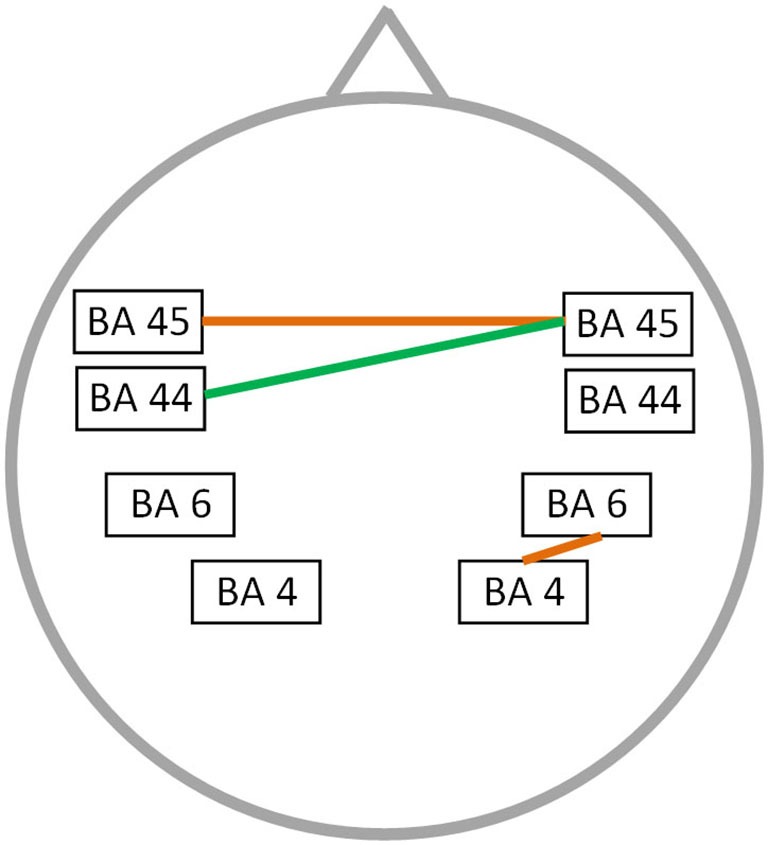
**Correlation between neural connectivity and TSS-R**. Positive correlation between neural connectivity and stuttering severity (*p* < 0.05) for the theta frequency band (orange lines) and the alpha 2 frequency band (green line).

A significant correlation was found between stuttering severity and neural connectivity for both the alpha 2 and theta frequency band (*r* = 0.76; *p* < 0.05) (Figure 3). For theta an increased synchronized activity is observed between left and right BA45 as well as between the right motor and premotor cortex. Furthermore, an increased connectivity between right BA45 and left BA44 is present for the alpha 2 frequency band. No significant correlation could be found for the delta, alpha 1, beta 1, beta 2, beta 3 and gamma frequency band.

### Functional connectivity: correlation with the impact of stuttering on daily living

**Figure 4:**
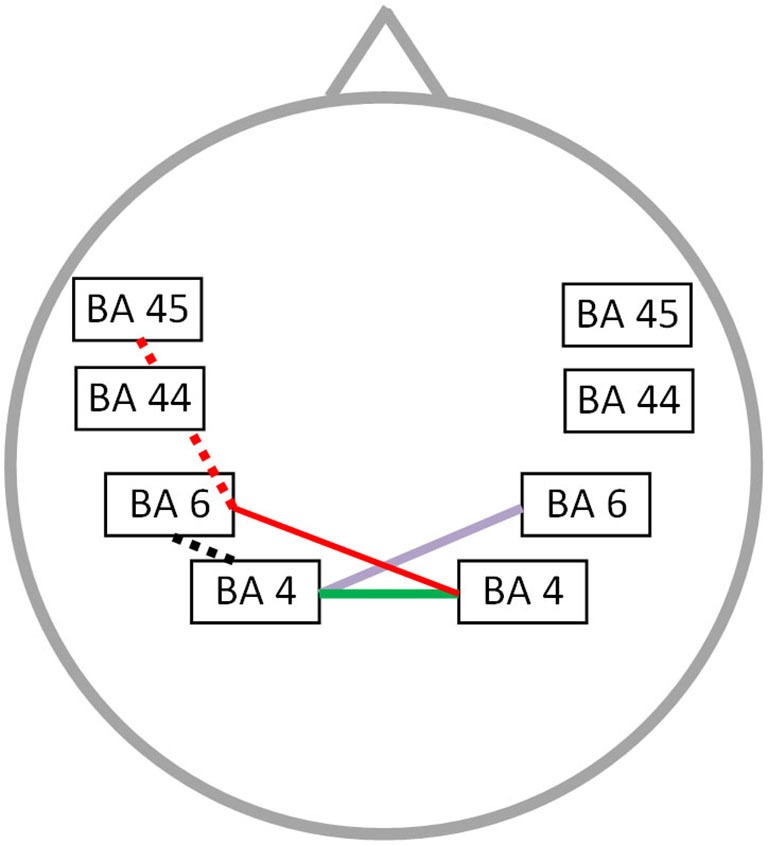
**Correlation between neural connectivity and OASES**. Positive correlation between neural connectivity and OASES (*p* < 0.05) for the delta frequency band (purple line), the alpha 1 and alpha 2 frequency band (green line) and the beta 1 frequency band (red line). A negative correlation was observed for the beta 1 frequency band (red dotted line) and the gamma frequency band (black dotted line).

The OASES correlated with neural connectivity in different frequency bands, including delta, alpha 1, alpha 2, beta 1 and gamma frequency band (*r* = 0.76; *p* < 0.05) (Figure 4). For delta an increased connectivity was present between left motor and right premotor area. For the alpha 1 and alpha 2 frequency band an increased synchronized activity between right and left motor cortices was present. Additionally, for the beta 1 frequency there is an increased connectivity between the right motor and left premotor cortex with a decreased functional connectivity between left premotor cortex and Broca’s area. We also identified an impaired connectivity between left motor and premotor cortex for the gamma frequency band. No significant correlation could be identified for the theta, beta 2 and beta 3 frequency bands.

### Frequency analysis of alpha rhythms

In addition, we calculated the individual alpha frequency (IAF) peak of all participants in order to be certain that our significant results were not due to differences in the IAF. The IAF peak was calculated according to literature guidelines (Klimesch, 1996, 1999). Subsequently we performed an independent samples *t*-test, which demonstrated no significant differences (*t*_(1,20)_ = 1.85; *p* = 0.08) in IAF between stuttering patients (*M* = 9.73; *SD* = 0.90) and controls (*M* = 9.04; *SD* = 0.82).

## Discussion

Previous studies mainly looked at structural changes or functional differences during speech-related tasks, but less is known about functional changes during resting state, reflecting the core neural aspects of stuttering. Furthermore, the limited studies are still inconsistent and further research is required.

A first important finding of this study is that no significant differences in resting state activity between PWS and controls could be found, neither in the whole brain analysis nor in the ROI analysis. In addition, no correlation could be demonstrated between neural activity and stuttering severity or QoL measures. This is in analogy to a previous PET study by Ingham et al. (1996), in which they could not identify significant changes of regional cerebral blood flow, although a previous PET study demonstrated relative flow asymmetry in the anterior cingulate cortex, superior and middle temporal gyri when comparing stuttering patients with fluently speaking controls (Pool et al., 1991). Recently, Xuan et al. (2012) investigated the amplitude of low-frequency alterations (ALFF) by resting state fMRI, a reflection of resting state local brain activity (Zang et al., 2007; Zuo et al., 2010), revealing an increased ALFF in the left IFG, while a decreased ALFF was found in bilateral supplementary motor areas and paracentral parietal lobes. These different results may be related to the application of diverse imaging techniques and the relatively small patient groups. In addition, although we could not observe significant results in resting state brain activity, we did identified significant differences in the theta and beta 1 frequency band when comparing the power spectra of both groups.

Contrary to the non-significant results when looking at resting state activity, we could obtain significant differences in brain connectivity of speech related areas between patients and fluently speaking controls. Currently, it is well known that a normal working brain requires the concerted action of multiple brain networks, hence necessitating an efficient communication between distinct brain areas. Moreover, in various pathologies, e.g., schizophrenia (Andreou et al., 2014) and Parkinson disease (Baggio et al., 2014), alterations in resting state functional connectivity have been revealed. The advantage of looking at resting state functional connectivity is that the observed alterations can rather be seen as inherent differences of a certain pathology rather than the consequence of a specific task or activity, e.g., stuttering speech. In addition, if impaired communication during resting state between distinct brain areas is already present, it is a natural consequence that the sequential activation of various brain regions during the performation of a complex task will not be optimal. First, we found a significantly decreased interhemispheric connectivity between speech-related brain areas for the beta 1, beta 3 and gamma frequency band. The decreased interhemispheric connectivity likely reflects the impaired communication of bilaterally located speech related motor areas. This could contribute to the phenomenology of stuttering as speech related muscles are bilaterally innervated and fluent speech requires a temporal synchronization of phonatory and articulatory muscle groups (Perkins et al., 1991). This is in accordance with the early theory of Orton and Travis in which they proposed that a disturbed cerebral dominance may have a causal impact on stuttering due to the conflicting activation of speech related brain areas (Orton, 1927; Orton and Travis, 1929). Comparing PWS with fluently speaking controls in a fMRI study (Xuan et al., 2012), demonstrated not only a decreased connectivity between the left IFG and right inferior parietal lobe, but also an increased functional connectivity between the left IFG and premotor cortex. Based on these results, they hypothesized that stuttering persons do not have the ability of increasing functional connectivity between the IFG and premotor cortex during speech as present in fluently speaking controls (Chang et al., 2011). Furthermore, Lu et al. demonstrated with fMRI a lower resting state functional connectivity of the left pars opercularis and a greater connectivity in the left part of the supplementary motor area and left cerebellum compared to fluently speaking controls (Lu et al., 2012). These observations confirm the hypothesis that stuttering has a more fundamental dysfunctional connectivity, not only present during speech or motor related tasks.

Besides the differences in resting state functional connectivity, we found a significant correlation between neural connectivity and stuttering severity for the theta and alpha 2 frequency bands. In both frequency bands we identified an increased resting state functional connectivity between left Broca’s area and its right sided homologue. Additionally an increased resting state functional connectivity was observed between the right motor and premotor cortex for the theta frequency band. These results are remarkable as previous studies mainly claimed that right sided hyperactivity in stuttering patients is related to an adaptive compensatory mechanism (Fox et al., 1996; Braun et al., 1997b; Preibisch et al., 2003). In contrast, some studies postulated that right sided lateralization is the representation of a maladaptive coping mechanism, i.e., a transient and overall insufficient repair process, as right sided increased activation is normalized by fluency shaping therapies (Fox et al., 1996; De Nil et al., 2003). The maladaptive compensation by the contralateral hemisphere may be in analogy with the results observed in unilateral stroke. Motor recovery can be impeded by hyperactivity of the contralateral motor cortex due to an increased interhemispheric inhibition by transcallosal neurons (Murase et al., 2004; Duque et al., 2005). In addition an optimal recovery from stuttering is related to white matter changes in the vicinity of the left rolandic operculum, indicating that the right hemisphere is not specialized enough for full recuperation (Kell et al., 2009). Moreover, structural alterations were found in the right hemisphere of stuttering persons, i.e., an increased sulcal connectivity with the right Sylvian fissure and a slight increase of folding in the right perisylvian region. These structural changes show a modest positive correlation with the stuttering severity as well (Cykowski et al., 2008). Making use of voxel-based morphometry an increased white matter volume could be identified for the right sided superior temporal gyrus, precentral gyrus, pars opercularis and middle frontal gyrus of PWS (Jäncke et al., 2004). Probably these changes are the result of long lasting alterations in functional connectivity. For example increased functional connectivity was identified between bilateral IFG, superior temporal gyri, angular gyri, cerebellum and right pars opercularis in stuttering persons when using a psychophysiological interaction (PPI) analysis on fMRI data (Chang et al., 2011). Moreover, Cykowksi et al. observed a decreased white matter integrity in the corpus callosum (Cykowski et al., 2008), potentially influencing interhemispheric communication of excitatory and inhibitory signals (Greiner et al., 1986). The impaired interhemispheric and right sided functional connectivity of speech related areas may be the result of the previous mentioned disconnection between left and right sided speech related brain areas when comparing controls to PWS, i.e., impaired communication of both hemispheres results in a maladaptive hyperactivity. The results of this study cannot give exclusion about the concept of right sided activity as adaptive or maladaptive, as we only looked at resting state functional connectivity, but it contributes to the interpretation of observed alterations during speech related tasks in PWS.

A significant correlation was found between neural functional connectivity and the total score of the OASES in different frequency bands, i.e., delta, alpha 1, alpha 2, beta 1 and gamma activity, as well as between stuttering severity and the OASES. This can be explained in two distinct ways, firstly is seems logical that patients stuttering more severely will experience a higher impact on their QoL, but it is plausible as well that persons more distressed by their stuttering have an aggravation of stuttering severity. However further research is necessary to give a clear explanation of our results.

To our knowledge this study is the first to look at brain activity and functional connectivity in PWS during resting state making use of sLORETA analysis, but some limitations have to be noticed. Firstly we only included 11 patients, and for only eight of them we could correlate neural activity/connectivity with the OASES questionnaire, as some of the questionnaires were not returned or not filled in by the patients. However, the major problem we were faced with is that adult stuttering patients are seldom still in therapy for their speech problems. In the future it will therefore be necessary to replicate this study in a larger group of stuttering persons.

A second limitation is that we included a limited number of ROI’s in our analyses. For subsequent research it might be interesting to look at a more widespread stuttering and distress network, but this was beyond the scope of our current study as we focused on the most consistently identified brain areas involved in stuttering. Otherwise, by making use of qEEG and sLORETA we eliminated one of the main limitations of previous studies making use of fMRI, i.e., the generation of a non-negligible amount of sound that influences neural activity.

In conclusion, the results of this study suggest that stuttering is not related to resting state activity changes in the brain, but it seems to be due to an impaired interhemispheric functional connectivity between motor speech areas, premotor and motor cortices in comparison to fluently speaking controls. Furthermore, stuttering persons severely affected by their stuttering show increased functional connectivity in speech related motor areas, possibly aggravating their stuttering severity and suggesting that the increased functional connectivity might be maladaptive.

## Conflict of interest statement

The authors declare that the research was conducted in the absence of any commercial or financial relationships that could be construed as a potential conflict of interest.
